# Mice lacking PLAP-1/asporin counteracts high fat diet-induced metabolic disorder and alveolar bone loss by controlling adipose tissue expansion

**DOI:** 10.1038/s41598-021-84512-2

**Published:** 2021-03-02

**Authors:** Hiromi Sakashita, Satoru Yamada, Masaki Kinoshita, Tetsuhiro Kajikawa, Tomoaki Iwayama, Shinya Murakami

**Affiliations:** 1 Department of Periodontology, Osaka University Graduate School of Dentistry, Suita, Osaka Japan; 2 Department of Periodontology and Endodontology, Tohoku University Graduate School of Dentistry, Sendai, Miyagi Japan; 3 Present Address: Department of Basic and Translational Sciences, Laboratory of Innate Immunity and Inflammation, Penn Dental Medicine, University of Pennsylvania, Philadelphia, PA USA

**Keywords:** Periodontitis, Obesity

## Abstract

Adipose tissue fibrosis with chronic inflammation is a hallmark of obesity-related metabolic disorders, and the role of proteoglycans in developing adipose tissue fibrosis is of interest. Periodontal disease is associated with obesity; however, the underlying molecular mechanisms remain unclear. Here we investigated the roles of periodontal ligament associated protein-1 (PLAP-1)/asporin, a proteoglycan preferentially and highly expressed in the periodontal ligament, in obesity-related adipose tissue dysfunction and adipocyte differentiation. It was found that PLAP-1 is also highly expressed in white adipose tissues. *Plap-1* knock-out mice counteracted obesity and alveolar bone resorption induced by a high-fat diet. *Plap-1* knock-down in 3T3-L1 cells resulted in less lipid accumulation, and recombinant PLAP-1 enhanced lipid accumulation in 3T3-L1 cells. In addition, it was found that primary preadipocytes isolated from *Plap-1* knock-out mice showed lesser lipid accumulation than the wild-type (WT) mice. Furthermore, the stromal vascular fraction of *Plap-1* knock-out mice showed different extracellular matrix gene expression patterns compared to WT. These findings demonstrate that PLAP-1 enhances adipogenesis and could be a key molecule in understanding the association between periodontal disease and obesity-related metabolic disorders.

## Introduction

Obesity is a major risk factor for type 2 diabetes and cardiovascular disease and is characterized by an excessive accumulation of adipose tissue. The expansion of adipose tissue is caused by proliferation and hypertrophy of adipocytes, which requires continuous remodeling of the extracellular matrix (ECM) to accommodate the expansion^1^. The flexibility of ECM enables healthy expansion of adipose tissue, and the decreased flexibility in the adipose tissue ECM is often associated with metabolically unhealthy obesity^2^. It is accepted that adipose tissue fibrosis is a major contributor to obesity-associated metabolic dysfunction^3^. Adipose tissue fibrosis may lead to apoptosis of adipocytes and chronic inflammation characterized by infiltrated macrophages^4^.

Although the deposition of fibrous collagen proteins such as collagen types I, III, and VI, is known to promote metabolic dysfunction in obesity^5^, the involvement of other ECM compartments, such as proteoglycans, is becoming increasingly evident^6^. The small leucine-rich proteoglycans (SLRPs) are the largest subfamily of proteoglycans and have been implicated in collagen fibrillogenesis^7^. Among the class I members of SLRP, opposing roles of Biglycan and Decorin in obesity and meta-inflammation are shown in KO mice models^8,9^. However, the other member of class I SLRP, PLAP-1/asporin, has not been investigated. PLAP-1 (Periodontal Ligament-Associated Protein-1) was identified in our laboratory in the human periodontal ligament (PDL) cDNA library^10,11^ and is preferentially and highly expressed in PDL tissue^12^. Because PLAP-1 has a pro-inflammatory effect on periodontal tissue by suppressing pathophysiologic TLR signaling^13^, we determined whether PLAP-1 is also involved in adipose tissue biology.

Periodontal disease is a chronic inflammatory disease that progressively destroys the periodontal apparatus, including the periodontal ligament and alveolar bone. Among the systemic diseases impacted by periodontal disease, obesity has been associated with an increased risk of periodontitis^14,15^. Consistent with epidemiologic evidence, high-fat diet (HFD) induced-obese mice have periodontitis^16^. The investigation of the association between obesity and periodontitis may help in understanding the mechanism of association and also improve knowledge of both diseases.

To elucidate the function and the effect of PLAP-1 in adipose tissue, we first showed *Plap-1* is expressed in adipose tissue, and the expression is down-regulated in long-term HFD-fed mice. *Plap-1* KO mice were protective to weight gain, adipose hypertrophy, and metabolic disorders upon HFD feeding compared to control mice, possibly through less macrophage infiltration in adipose tissue. Furthermore, *Plap-1* KO mice showed less HFD-induced alveolar bone resorption. In parallel with these in vivo analyses, in vitro experiments both by silencing *Plap-1* and by treatment with recombinant PLAP-1 demonstrated that PLAP-1 promotes adipocyte differentiation of preadipocyte cell line, 3T3-L1 cells. The finding was further confirmed in the primary culture preadipocytes from *Plap-1* KO mice. In addition, *Plap-1* KO mice showed different gene expressions of ECM in adipose tissue from wild-type (WT). Collectively, these data suggest that PLAP-1 has a protective role in HFD-induced metabolic disorder and alveolar bone loss by controlling adipose tissue expansion.

## Materials and methods

### Animals

All animal experiments were approved by the Institutional Animal Care and Use Committee of Osaka University Graduate School of Dentistry and complied with the guidelines for the care and use of laboratory animals at Osaka University. We also pledge that this study was carried out in compliance with the ARRIVE guidelines appropriately. Mice with HFD-induced obesity were established based on a previously published method^17^. We fed 5-week-old WT and *Plap-1* knock-out (*Plap-1* KO) mice that were generated in our laboratory^18^, with 60 kcal % HFD [20% kcal protein, 60% kcal fat, 20% kcal carbohydrate, and 5.21 kcal/g energy density] (Research Diets, New Jersey, USA) or 10 kcal % normal chow diet (NC) [20% kcal protein, 10% kcal fat, 70% kcal carbohydrate and 3.82 kcal/g energy density] (Research Diets) as the control diet. Food intake per cage per week was measured.

### RNA extraction and quantitative polymerase chain reaction analysis

Maxilla, white adipose tissue (WAT), brown adipose tissue, brain, heart, lung, liver, stomach, small intestine, large intestine, pancreas, kidney, spleen, bone marrow, muscle, and gingiva were extracted from 8-week-old male WT mice. WAT was extracted from WT and *Plap-1* KO mice after 16 weeks of feeding NC or HFD. Total RNA was extracted from these tissues and cultured cells using the RNeasy Lipid Tissue Mini Kit (QIAGEN, Hilden, Germany) or PureLink RNA Mini Kit (Life Technologies, California, USA), respectively. Total RNA was reverse transcribed to cDNA using the High Capacity RNA-to-cDNA Kit (Applied Biosystems, California, USA), and quantitative polymerase chain reaction (PCR) was performed with the StepOnePlus Real-time PCR System (Applied Biosystems) using Fast SYBR Green Master Mix and gene-specific primers (Table S1).

### Glucose metabolic analysis

Before and after feeding NC or HFD, the glucose tolerance test (GTT) and insulin tolerance test (ITT) were performed. For the GTT, mice were starved for 16 h and were injected with 1.5 g kg^−1^ (bodyweight) glucose (Wako Pure Chemical Industries, Osaka, Japan) intraperitoneally. For the ITT, mice were injected with 0.75 U kg^−1^ (bodyweight) human insulin (Novo Nordisk, Bagsvaerd, Denmark) intraperitoneally without starvation. Tail vein blood was collected at 0, 15, 30, 60, and 120 min after administration to measure blood glucose levels with ONE TOUCH Ultra (Johnson & Johnson Services, New Jersey, USA).

### Biochemical serum marker test

After 16 h of starvation, blood was collected from mice, incubated for 1 h at room temperature, and then centrifuged (1,500 g, room temperature, 15 min) to obtain serum. Serum total cholesterol (T-CHO), triglyceride (TG), non-esterified fatty acids (NEFA), low-density lipoprotein cholesterol (LDL-C), high-density lipoprotein cholesterol (HDL-C), glucose concentrations, insulin, and adiponectin were determined using L-type Wako CHO-H (Wako Pure Chemical Industries, Osaka, Japan), L-type Wako TG-H (Wako Pure Chemical Industries, Osaka, Japan), NEFA-SS Eiken (Eiken Chemical, Tochigi, Japan), Cholestest LDL (SEKISUI MEDICAL, Tokyo, Japan), Cholestest N HDL (SEKISUI MEDICAL), Quick Auto Neo GLU-HK (Sino Test, Tokyo, Japan), Ultra Sensitive Mouse Insulin ELISA kit (Morinaga Institute of Biological Science, Kanagawa, Japan) and Mouse/Rat adiponectin ELISA kit (Otsuka Pharmaceutical, Tokyo, Japan) respectively.

### Quantitative analysis of alveolar bone resorption

Maxilla was collected from WT and *Plap-1* KO mice at the start point (5 week-old) and after feeding NC or HFD for 16 weeks (21 week-old) and imaged by 3D micro X-ray CT R_mCT2 (Rigaku, Tokyo, Japan). These images were analyzed using TRI/3D-BON software (RATOC SYSTEM ENGINEERING, Tokyo, Japan). Distance between the alveolar bone crest and the cement-enamel junction in both the buccal and palatal side was measured at the distal root of the first molar and mesial and distal roots of the second molar in the apical direction using WinROOF software (Mitani Corporation, Fukui, Japan). The total value of these three distances was considered as the alveolar bone resorption.

### Quantitative analysis of adipose tissue

WT and *Plap-1* KO mice after feeding HFD for 16 weeks were imaged by 3D micro X-ray CT R_mCT2 and analyzed using CTAtlas Metabolic Analysis Ver. 2.03 software (Rigaku).

### Histological analysis

Epididymal adipose tissue was collected from WT and *Plap-1* KO mice after 16 weeks of feeding HFD and then fixed in 4% Paraformaldehyde Phosphate Buffer Solution (Wako Pure Chemical Industries, Osaka, Japan) overnight. Samples were embedded in paraffin and sectioned at 3.0 μm with LEICA RM2245 (Leica Microsystems, Wetzlar, Germany). Sections were stained with Mayer’s Hematoxylin (MUTO PURE CHEMICALS, Tokyo, Japan) and 1% Eosin Y Solution (Wako Pure Chemical Industries, Osaka, Japan). Stained sections were observed and imaged with ECLIPSE Ci (Nikon, Tokyo, Japan). The size of adipocytes was measured with ImageJ software.

### 3T3-L1 cell culture

3T3-L1 cells in Dulbecco’s Modified Eagle’s Medium (D-MEM; Life Technologies, California, USA) supplemented with 10% fetal bovine serum (FBS; Life Technologies, California, USA) without antibiotics were seeded at 4 × 10^4^ cells per well in a 24-well cell culture plate (Corning, New York, USA) and 2 × 10^5^ cells per well in a 6-well cell culture plate (Corning, New York, USA), and then transfected with Silencer Select *Plap-1* siRNA (Assay ID: s83722) or Silencer Select Negative Control siRNA (Life Technologies, California, USA) through Lipofectamine 3000 (Life Technologies, California, USA). Six hours after transfection, the medium was exchanged with D-MEM supplemented with 10% FBS and 60 μg/ml of kanamycin. Seventy-two hours after cell seeding, 3T3-L1 cells were incubated for 48 h with adipocyte induction medium containing 0.5 mM isobutyl-methylxanthine (IBMX; Sigma-Aldrich, Missouri, USA), 1 μM dexamethasone (DEX; Sigma-Aldrich, Missouri, USA), and 10 μg/ml insulin (Sigma-Aldrich, Missouri, USA) to induce adipocyte differentiation, and then cultured with adipocyte maintenance medium containing 10 μg/ml insulin to maintain adipocyte differentiation for another 9 days (changed media every 2 days). Total RNA extraction and Oil Red O staining were performed with the cells seeded in 6- and 24-well cell culture plates, respectively.

### Conditioned medium containing recombinant PLAP-1

3T3-L1 cells were infected with adenoviruses that carried *LacZ* or FLAG-tag mouse *Plap-1*^18^, and the supernatant was collected as the conditioned medium (CM) after 48 h. To confirm the expression of PLAP-1 in the CM, cultured cell supernatants (24 μl) were separated by 10% sodium dodecyl sulfate–polyacrylamide gel electrophoresis and subjected to Western blotting. Horseradish peroxidase (HRP)-linked mouse anti-FLAG antibody (1:10,000; Sigma-Aldrich, Missouri, USA) was used for the detection of PLAP-1. Immunoreactive proteins were visualized by SuperSignal West Dura Extended Duration Substrate (Thermo Scientific, Illinois, USA) with the ImageQuant LAS4000 imager (GE Healthcare, New Jersey, USA). The membrane was incubated in Ponceau S solution (Sigma-Aldrich, Missouri, USA) for 10 min and imaged after washing with water for 5 min.

### Adipocyte differentiation utilizing PLAP-1 CM

3T3-L1 cells in D-MEM supplemented with 10% FBS and 60 μg/ml of kanamycin were seeded at 2 × 10^4^ cells per well and 8 × 10^4^ cells per well in 24- and 6-well fibronectin-coated cell culture plates, respectively. Four days after seeding, confluent 3T3-L1 cells were cultured with adipocyte induction medium described above with or without 100% PLAP-1 CM for 48 h, and then cultured with adipocyte maintenance medium with or without 100% PLAP-1 CM. Total RNA extraction and Oil Red O staining were performed with cells seeded in 6- and 24-well cell culture plates, respectively.

### Isolation of primary preadipocytes and adipocyte differentiation

Primary preadipocyte isolation was performed according to a previously published protocol^19^ with modifications. Minced adipose tissue from WT and *Plap-1* KO mice was digested in D-MEM supplemented with 4000 units/ml type II collagenase from *Clostridium histolyticum* (Sigma-Aldrich, Missouri, USA), 0.1 mg/ml DNase I (Roche, Basel, Switzerland), 10% FBS and 60 μg/ml of kanamycin at 37ºC for 30 min. The digest was filtrated with 40 μm Cell Strainer (BD Biosciences, California, USA) and centrifuged (500 g, room temperature, 10 min). The floating layer and cell pellet were obtained as mature adipocyte fraction (MAF) and stromal vascular fraction (SVF), respectively. SVF was suspended in D-MEM supplemented with 10% FBS and 60 μg/ml of kanamycin and seeded in 24- and 6-well cell culture plates. On the next day, cells were rinsed three times with phosphate-buffered saline and cultured until reaching confluence. Cells were incubated with 10 μM pioglitazone (Sigma-Aldrich, Missouri, USA), 0.5 mM IBMX, 1 μM DEX, and 1 μM insulin for 48 h to induce adipocyte differentiation and then cultured with 100 nM insulin to maintain adipocyte differentiation. Total RNA extraction and Oil Red O staining were performed with the cells seeded in 6- and 24-well cell culture plates, respectively.

### Oil Red O staining and quantitative lipid assay

Lipid accumulation in 3T3-L1 cells or preadipocytes isolated from mice was detected by Oil Red O staining utilizing Lipid Assay Kit (Cosmo Bio, Hokkaido, Japan). Cells were rinsed with phosphate-buffered saline and fixed in 10% Formaldehyde Solution (Wako Pure Chemical Industries) overnight. The fixed cells were washed with purified water and stained with Oil Red O solution for 15 min. After three washes, cells in purified water were imaged with ECLIPSE Ti (Nikon). For the quantification, isopropanol was applied to the stained cells, and absorbance at 540 nm of the extracted solution was measured with the Multiskan FC Microplate Photometer (Thermo Scientific, Illinois, USA).

### Statistical analysis

Data were described using mean ± standard deviation. Statistical analyses were performed using the Student’s t-test for paired comparisons and one-way analysis of variance for multiple comparisons with Tukey’s post hoc test. A value of *p* < 0.05 was considered statistically significant. All data were analyzed using GraphPad Prism.

## Results

### Plap-1 is expressed in adipose tissue and downregulated in obesity

**Figure 1:**
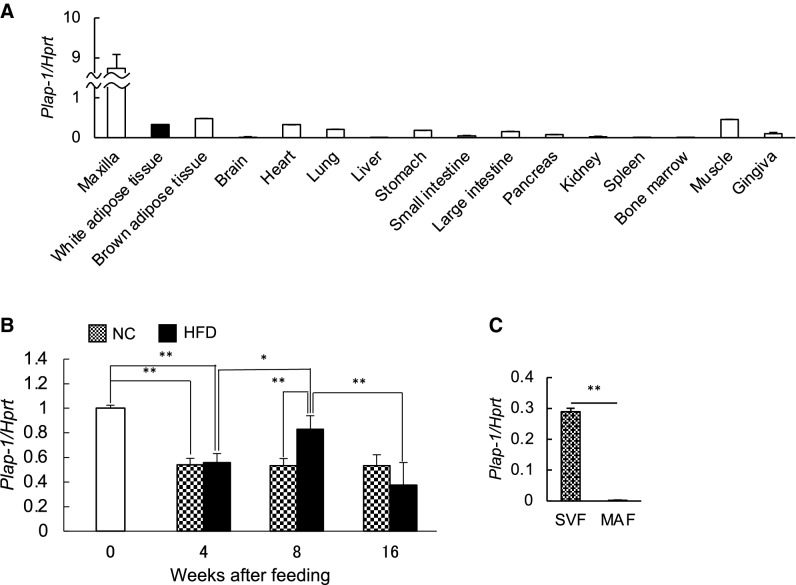
PLAP-1 is expressed in adipose tissue. (**A**)Total RNA was extracted from various tissues and the expressions of *Plap-1* were analyzed by real-time PCR analysis. Results show the mean ± SD of triplicate assays. (**B**) *Plap-1* expression in epididymal adipose tissue was analyzed by real-time PCR analysis before or during NC or HFD feeding. 0: start point of NC or HFD feeding (five weeks of age) (*n* = 3), 4: after 4 weeks NC or HFD feeding (*n* = 5 in each group), 8: after 8 weeks NC or HFD feeding (*n* = 5 in each group), 16: after 16 weeks NC or HFD feeding (*n* = 5 in each group). (**C**) Real-time PCR analysis was performed to investigate *Plap-1* expression in SVF and MAF isolated from WT and *Plap-1* KO mice. Values represent the mean ± SD triplicate assays.

We have previously demonstrated that PLAP-1 is preferentially and highly expressed in PDL and has important roles in maintaining periodontal homeostasis by suppressing BMP-2 and TGF-β signaling. We analyzed *Plap-1* expression in other tissues and organs in mice and found that *Plap-1* was also expressed in WAT (Fig. 1A). Thus, we hypothesized that PLAP-1 might have some crucial roles in WAT. To investigate the role of PLAP-1 in adipose and periodontal tissues, we fed mice with HFD and measured metabolic parameters. The mice fed with HFD had significantly greater body weight as compared to those fed with NC (Fig. S1A). In addition, they showed impaired glucose tolerance and insulin resistance after 16 weeks of HFD feeding (Fig. S1B). Furthermore, they showed significantly higher serum levels of total cholesterol, HDL-C, glucose, and lower serum level of triglyceride than NC-fed mice (Fig. S1C). Furthermore, we assessed alveolar bone resorption by micro-computed tomography (μCT) analysis. The NC-fed mice showed no significant alveolar bone resorption during 16 weeks of feeding. However, HFD-fed mice had more alveolar bone resorption than NC-fed mice significantly at 16 weeks of feeding (Fig. S2A, B). To analyze the expression of *Plap-1* in epididymal adipose tissue during HFD feeding, we performed real-time PCR analysis. *Plap-1* expression was high at the start point of NC or HFD feeding, that is, at five weeks of age (Fig. 1B). Afterward, *Plap-1* expression decreased in mice fed with NC and increased after eight weeks of HFD feeding (Fig. 1B). To understand which cell types in the adipose tissue mainly express *Plap-1*, we separated subcutaneous adipose tissue into MAF and SVF. Interestingly, *Plap-1* expression was predominant in SVF (Fig. 1C), suggesting that PLAP-1 expressing cells are mainly preadipocytes and fibroblasts, but not adipocytes.

### Genetic ablation of *Plap-1* protects mice from HFD-induced over-weight and hypertrophy of adipocytes

**Figure 2:**
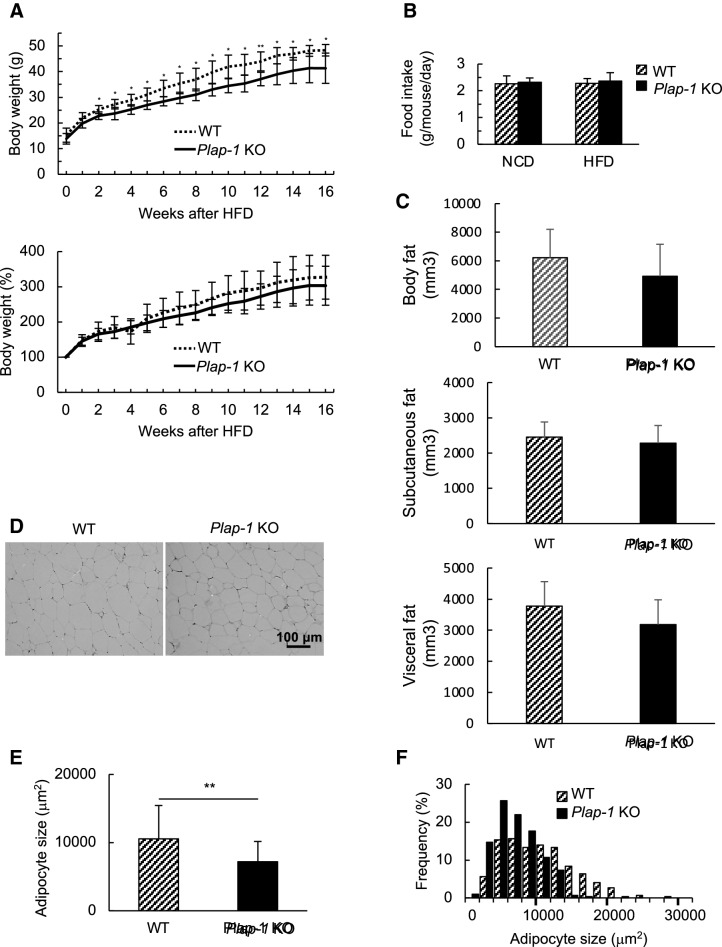

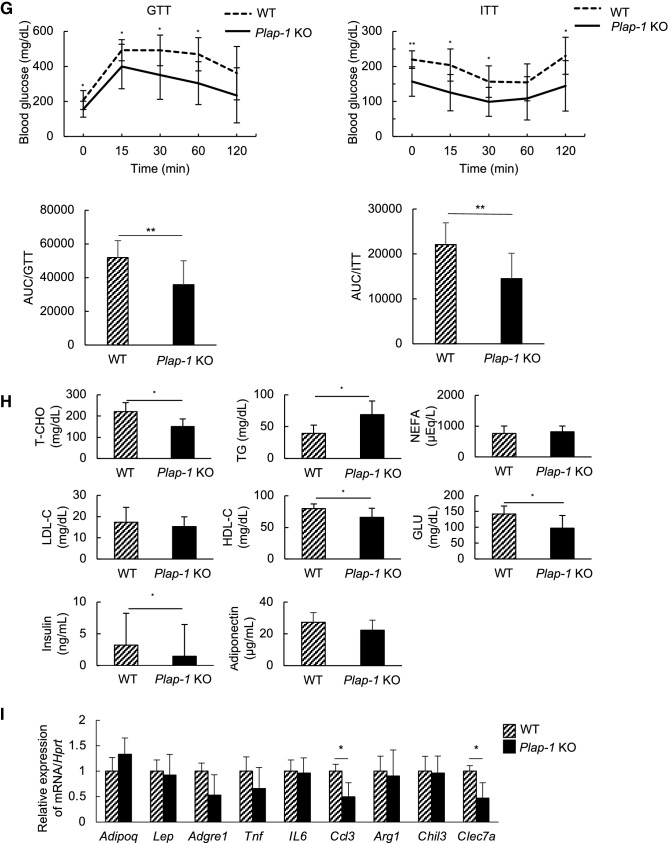
Body weight and expansion of adipocytes in *Plap-1* KO mice after HFD feeding. (**A**) Body weight changes in WT and *Plap-1* KO mice during HFD feeding. 5-week-old male WT and *Plap-1* KO mice were fed with HFD and weighted weekly. WT (n = 7), *Plap-1* KO (n = 9). Both g body weight and % body weight are shown. Results show the mean ± SD. *: *p* < 0.05, **: *p* < 0.01 (**B**) Food intake per mouse per day during NC or HFD feeding were measured (n = 4 in each group). Results show the mean ± SD. (**C**) H-E staining of epididymal adipose tissue was conducted in WT and *Plap-1* KO mice after 16 weeks of HFD feeding (× 50). (**D** and **E**) Average adipocyte size (**D**) and frequency of adipocyte size (**E**) of epididymal fat were shown. To analyze adipocyte size, **H–E** staining sections were obtained from three mice per each group, and the area of 100 adipocytes in each mouse was measured. Results show the mean ± SD. **: *p* < 0.01 (**F**) GTT and ITT were performed in WT and *Plap-1* KO mice after 16 weeks of HFD feeding. WT (n = 10), *Plap-1* KO (n = 11). Results show the mean ± SD. *: *p* < 0.05, **: *p* < 0.01 (**G**) Metabolic serum markers in WT and *Plap-1* KO mice after 16 weeks HFD feeding. Serum levels of total cholesterol (T-CHO), triglyceride (TG), LDL-cholesterol (LDL-C), HDL-cholesterol (HDL-C), non-esterified fatty acid (NEFA), glucose (GLU), insulin, and adiponectin were measured after HFD feeding. WT (n = 6), *Plap-1* KO (n = 11). Results show the mean ± SD. (**H**) Real-time PCR analysis was performed in epididymal adipose tissue of WT and *Plap-1* KO mice after 16 weeks of HFD feeding. WT (n = 4), *Plap-1* KO (n = 5). Data are represented as relative expression to WT mice. Results show the mean ± SD. *: *p* < 0.05.

To investigate the functional involvement of PLAP-1 in adipose tissue homeostasis in a physiological condition, we first compared *Plap-1* KO mice to WT during NC feeding. We found that *Plap-1* KO mice tended to gain less body weight than WT, but it did not reach statistical significance (Fig. S3A). GTTs and ITTs at the basal state (5-week-old) were comparable between *Plap-1* KO and WT mice, although there was a significant difference in GTT after 16 weeks of NC feeding (Fig. S3B, C). These data suggest that *Plap-1* KO mice are resistant to metabolic disorders. To study the roles of PLAP-1 under HFD conditions, we fed WT and *Plap-1* KO mice with HFD and measured their metabolic parameters. *Plap-1* KO mice counteracted HFD-induced overweight (Fig. 2A), although there was no difference in the food intake between WT and *Plap-1* KO mice (Fig. 2B). Both subcutaneous and visceral fat mass was lower in *Plap-1* KO mice, although the difference did not reach statistical significance (Fig. 2C). Based on the histomorphological quantification, we detected that the average size of an adipocyte was decreased, and the number of small adipocytes was increased in *Plap-1* KO mice (Fig. 2D–F). From these data, we concluded that hypertrophy in *Plap-1* KO mice was decreased.

### *Plap-1* deficiency ameliorates HFD-induced metabolic disorders

There were no statistical differences of GTT and ITT between WT and *Plap-1* KO mice when they were fed with NC (Fig. S3B). However, *Plap-1* KO mice showed high glucose tolerance and enhanced insulin sensitivity after 16 weeks of HFD feeding (Fig. 2G). We performed biochemical serum maker tests after 16 weeks of HFD feeding to investigate whether PLAP-1 has any influence on metabolic serum markers. Total cholesterol and glucose levels of *Plap-1* KO mice were significantly lower than that of WT mice. On the other hand, the triglyceride level was higher than that of WT mice. These results suggest that *Plap-1* KO mice may have a low lipoprotein lipase (LPL) activity, which decomposes triglyceride in the serum to free fatty acid and glycerol (Fig. 2H). Furthermore, insulin level was lower in *Plap-1* KO mice, although there was no difference in adiponectin (Fig. 2H). These data show that the deletion of *Plap-1* improves metabolic disorder under HFD conditions.

### Macrophage marker gene expressions are suppressed in adipose tissue of *Plap-1* KO mice

We then analyzed adipokine expression in epididymal adipose tissue using real-time PCR. *Adipoq*, which exhibits a protective role in HFD-induced insulin resistance^20^, was relatively highly expressed in *Plap-1* KO mice, but the expression did not reach statistical significance (*p* = 0.148), and there was no difference in the expression of *Lep* (Fig. 2I). It is well known that macrophages infiltrate into adipose tissue in HFD fed mice, and thus we also examined the gene expressions for macrophage markers. We found that *Adgre1*, which encodes F4/80, was expressed lower in *Plap-1* KO mice (Fig. 2I), although the difference did not reach statistical significance (*p* = 0.064). To characterize infiltrated macrophages in adipose tissue, we assessed expressions of M1 and M2 macrophage markers. M1 macrophage markers, *Ccl3*, and an M2 macrophage marker, *Clec7a*^21^, were statistically lower in *Plap-1* KO mice than in WT mice (Fig. 2I). These results suggest less macrophage infiltration in WAT of *Plap-1* KO mice than the WT.

### *Plap-1* KO mice show less HFD-induced alveolar bone resorption

**Figure 3:**
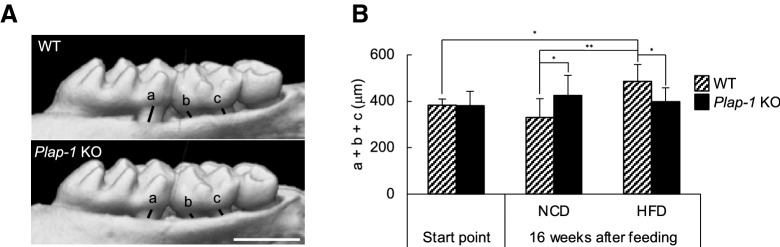
Evaluation of HFD-induced alveolar bone resorption. (**A**) Alveolar bone resorption was evaluated by μCT analysis in WT and *Plap-1* KO mice fed with HFD. (**B**) Distance between alveolar bone crest and cement-enamel junction was measured at distal root of first molar (a), mesial (b) and distal (c) root of second molar. Start point/WT (n = 10), start point/*Plap-1* KO (n = 10), 16 weeks after feeding/NC/WT (n = 10), 16 weeks after feeding/NC/*Plap-1* KO (n = 8), 16 weeks after feeding/HFD/WT (n = 10), 16 weeks after feeding/HFD/*Plap-1* KO (n = 10). Results show the mean ± SD. *: *p* < 0.05, **: *p* < 0.01.

To investigate the involvement of PLAP-1 in periodontal health associated with HFD feeding, we assessed alveolar bone resorption of *Plap-1* KO mice by μCT analysis (Fig. 3A). After 16 weeks of HFD feeding, alveolar bone resorption of *Plap-1* KO was significantly less than WT mice (Fig. 3B), suggesting that *Plap-1* KO mice were protective against HFD-induced alveolar bone resorption.

### Endogenous PLAP-1 inhibits adipocyte differentiation in 3T3-L1 cells

**Figure 4:**
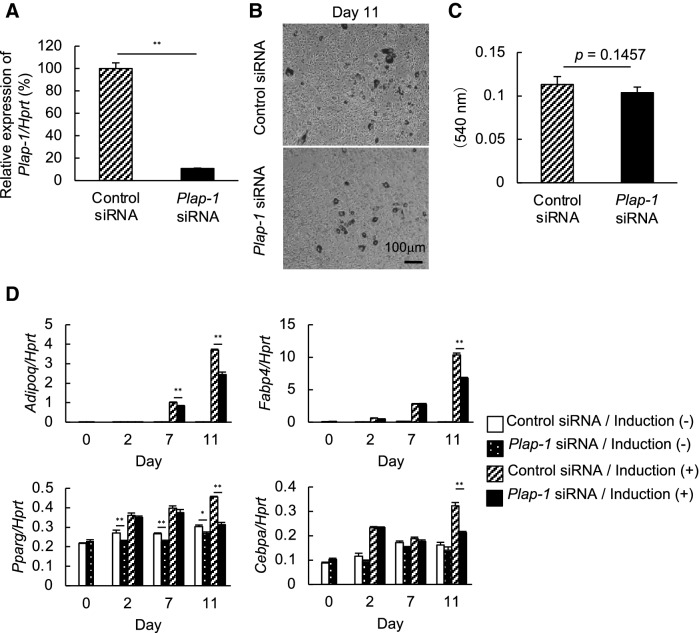
Adipocyte differentiation of *Plap-1* knock-down 3T3-L1 cells by siRNA. (**A**) Real-time PCR analysis was performed to confirm *Plap-1* knock-down by siRNA in 3T3-L1 cells. Data are represented as relative expression to control siRNA. Values represent the mean ± SD triplicate assays (n = 3). (**B**) Oil Red O staining of *Plap-1* knock-down and control 3T3-L1 cells was conducted after adipocyte differentiation. (**C**) Quantitative lipid assay of *Plap-1* knock-down and control 3T3-L1 cells was performed after Oil Red O staining (*n* = 4 in each group). Values represent the mean ± SD (n = 4). (**D**) Real-time PCR analysis was performed to examine adipogenic gene expressions of *Plap-1* knock-down 3T3-L1 cells during adipocyte differentiation. Results show the mean ± SD of triplicate assays (n = 3). *: *p* < 0.05, **: *p* < 0.01.

To further understand how PLAP-1 affects adipocyte differentiation, we utilized 3T3-L1 preadipocyte. We inhibited the endogenous expression of Plap-1 in 3T3-L1 cells by siRNA (Fig. 4A) and induced adipocyte differentiation using these cells. Oil Red O staining showed less lipid accumulation in Plap-1 knocked-down 3T3-L1 cells (Fig. 4B, C). Furthermore, the real-time PCR analysis revealed that Adipoq, Fabp4, Pparg, and Cebpa were significantly down-regulated in Plap-1 knocked-down 3T3-L1 cells (Fig. 4D). These results suggest that the endogenous PLAP-1 expressed by preadipocyte promotes its differentiation in a cell-autonomous manner.

### PLAP-1 promotes adipocyte differentiation in 3T3-L1 cells

**Figure 5:**
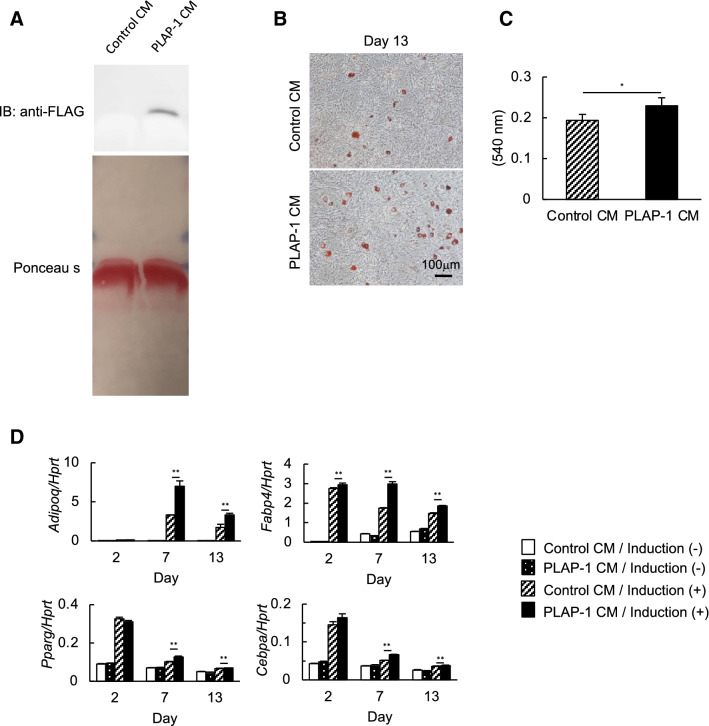
Adipocyte differentiation of 3T3-L1 cells in the presence of recombinant PLAP-1. (**A**) PLAP-1 expression in culture supernatants from 3T3-L1 cells infected with adenoviruses that carried *LacZ* or mouse *Plap-1* was identified by Western blot analysis. Total protein level was shown by Ponceau S. Control CM: Control conditioned medium infected with adenoviruses that carried *LacZ*, PLAP-1 CM: PLAP-1 conditioned medium infected with adenoviruses that carried *Plap-1*. The original blot images are included in Fig. S4. (**B**) Oil Red O staining of 3T3-L1 cells in the presence of recombinant PLAP-1 was conducted after adipocyte differentiation. (**C**) Quantitative lipid assay of 3T3-L1 cells in the presence of recombinant PLAP-1 was performed after Oil Red O staining (*n* = 4). Values represent the mean ± SD. (**D**) Real-time PCR analysis was performed to examine adipogenic related gene expression of 3T3-L1 cells in the presence of recombinant PLAP-1 during adipocyte differentiation (n = 3). Results show the mean ± SD of triplicate assays. *:*p* < 0.05, **: *p* < 0.01.

To investigate if exogenous PLAP-1 can promote adipocyte differentiation, we induced adipocyte differentiation of 3T3-L1 cells in the presence of recombinant PLAP-1. We induced adipocyte differentiation in the presence of PLAP-1 conditioned medium (PLAP-1 CM) obtained from PLAP-1-overexpressing 3T3-L1 cells. We confirmed PLAP-1 expression in PLAP-1 CM by Western blot analysis (Fig. 5A). Oil Red O staining and quantitative lipid assay demonstrated that lipid accumulation in the presence of PLAP-1 was significantly enhanced compared to the control (Fig. 5B, C). Furthermore, *Adipoq*, *Fabp4*, *Pparg*, and *Cebpa* expressions were increased in the presence of recombinant PLAP-1 (Fig. 5D). These results demonstrated that exogenous PLAP-1 could rigorously promote adipocyte differentiation.

### Genetic depletion of *Plap-1* inhibits adipocyte differentiation of preadipocytes isolated from mice

**Figure 6:**
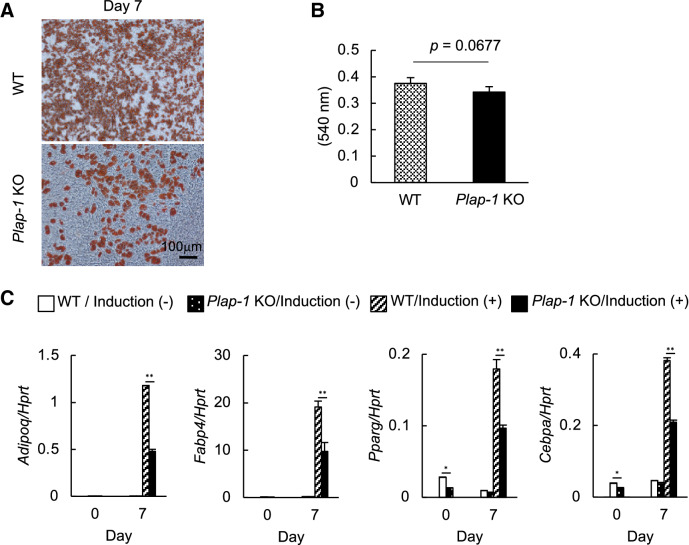
Adipocyte differentiation of primary preadipocytes isolated from WT and *Plap-1* KO mice. (**A**) Oil Red O staining of SVF isolated from WT and *Plap-1* KO mice was conducted after adipocyte differentiation. (**B**) Quantitative lipid assay of SVF isolated from WT and *Plap-1* KO mice was performed after Oil Red O staining (n = 4 in each group). Values represent the mean ± SD. (**C**) Real-time PCR analyses were performed to examine adipogenic related gene expression of SVF isolated from WT and *Plap-1* KO mice during adipocyte differentiation. Results show the mean ± SD of triplicate assays. n = 3, *: *p* < 0.05, **: *p* < 0.01.

We isolated primary preadipocytes from subcutaneous adipose tissue of WT and *Plap-1* KO mice and induced adipocyte differentiation to investigate whether adipocyte differentiation in *Plap-1* KO mice is also decreased in vivo. As we described, *Plap-1* expression was higher in SVF than in MAF (Fig. 1C), and therefore we utilized primary preadipocytes obtained from SVF. In accordance with the 3T3-L1 results, preadipocytes from *Plap-1* KO mice showed less lipid accumulation (Fig. 6A, B). Furthermore, preadipocytes from *Plap-1* KO mice showed low expressions of *Adipoq*, *Fabp4*, *Pparg,* and *Cebpa* than WT cells (Fig. 6C). These results indicate that adipocyte differentiation was significantly suppressed in primary preadipocytes isolated from *Plap-1* KO mice.

### *Plap-1* KO mice show different gene expressions of ECM in adipose tissue from WT

**Figure 7:**
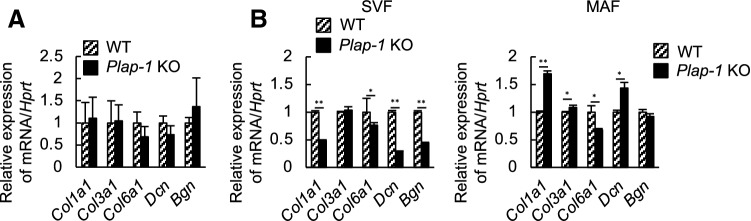
ECM gene expressions in adipose tissue. (**A**) ECM gene expressions in subcutaneous adipose tissue of WT and *Plap-1* KO mice were investigated by real-time PCR analysis. (**B**) ECM gene expressions in SVF and MAF isolated from WT and *Plap-1* KO mice were investigated by real-time PCR analysis. All data are represented as relative expression to WT. Col1a1: collagen, type I, alpha 1, Col3a1: collagen, type III, alpha 1, Col6a1: collagen, type VI, alpha 1, Dcn: Decorin, Bgn: Biglycan. Results show the mean ± SD of triplicate assays. n = 3, *: *p* < 0.05, **: *p* < 0.01.

ECM surrounding adipocytes serves as a mechanical support and has a vital role in maintaining homeostasis in adipose tissue^22,23^. It has been reported that the expansion of adipose tissue increased ECM gene expressions, and excessive ECM accumulation, especially of collagen I, III, and VI, caused fibrosis^22^. We sought the possibility that PLAP-1 affects the expression of other ECMs that have pivotal roles in adipose tissue fibrosis. Whole adipose tissue, SVF, and MAF were obtained from WT and *Plap-1* KO mice, and ECM gene expression was investigated. There was no difference in ECM gene expressions in whole adipose tissue of *Plap-1* KO and WT mice (Fig. 7A). However, the expression of *Col1a1 Col6a1*, *Dcn*, and *Bgn* in SVF of *Plap-1* KO mice was significantly lower than those of WT. Furthermore, *Col1a1* and *Dcn* expressions were higher, and that of *Col6a1* and *Bgn* were lower in MAF of *Plap-1* KO mice than WT (Fig. 7B). These results demonstrated that ECM gene expression patterns in adipose tissue are different between WT and *Plap-1 KO* mice and could be involved in the enhancement of fibrosis in adipose tissue.

## Discussion

In this study, we investigated the HFD-induced metabolic change in *Plap-1* KO mice and assessed the effect of PLAP-1 on adipocyte differentiation in preadipocytes. We demonstrated that PLAP-1 is expressed not only in PDL tissue but also in adipose tissue (Fig. 1A) and that it regulates the development of HFD-induced metabolic disorder and alveolar bone resorption (Figs. 2 and 3). We also found that PLAP-1 promotes adipocyte differentiation in preadipocytes. Furthermore, we showed that PLAP-1 is involved in the expression of ECM, which has essential roles in the fibrosis of adipose tissue.

Obesity is one of the primary causes of diabetes, which is characterized by insulin resistance. Adipocytes are surrounded by ECM, and the flexibility of ECM organized by collagen fibrillogenesis allows healthy expansion (hyperplasia) of visceral adipose tissue, which is in an anti-inflammatory state. However, when adipose tissue expands in an unhealthy manner (hypertrophy), it is in a pro-inflammatory state and leads to insulin resistance and fibrosis^24^. It has been revealed that HFD causes insulin resistance, high concentrations of total cholesterol and glucose, and develops obesity in mice^25–27^. Systemic glucose homeostasis correlates with subcutaneous WAT fibrosis and visceral adiposity^28^. In our study, we established the obesity model mice by HFD feeding, and they exhibited insulin resistance and hyperglycemia. SLRPs regulate collagen fibrillogenesis, possibly through their binding sites to collagen fibril. Type I SLRP members, Biglycan, Decorin, and PLAP-1/Asporin, show high structural similarity, although each SLRP has a different role in collagen fibrillogenesis^7^. The role of Biglycan and Decorin in adipose tissue biology has been reported^8,9^; however, that of PLAP-1 has not been elucidated yet. We found that *Plap-1* expression in adipose tissue was high in 5-week-old WT mice fed with NC and decreased as they grew old. These results suggest a possible involvement of PLAP-1 in adipose tissue homeostasis and healthy expansion. The rapid expansion of adipose tissue causes eventually leads to fibrosis due to excessive expression of ECM. In our study, *Plap-1* expression was significantly increased in adipose tissue of WT mice after eight weeks of HFD feeding. These results suggest that PLAP-1 may be induced and contribute to fibrotic changes in adipose tissue. Hypertrophy of adipocytes induced by HFD in *Plap-1* KO mice was inhibited compared to WT mice, and one of the reasons for the inhibitory effect could be the influence on ECM flexibility via collagen fibrillogenesis. Under NC condition, collagen amount and capillary density in adipose tissue of *Plap-1* KO mice were comparable to WT (Trichrome and GS-IB4 staining, respectively). There was no difference in the expression of macrophage markers (F4/80 and CD68) and other type I SLRP members (Biglycan and Decorin) between these mice (data not shown). The inhibitory effect of PLAP-1 in the development of obesity might be prominent in the unhealthy adipose expansion. We will investigate the more detailed mechanism of how PLAP-1 affects obesity and metabolic disorders in future study. The adipose depot specificity is another important topic in future study since adipocytes have diverse origin and mechanism of differentiation depends on adipose depots^29^.

It has been reported that obesity and metabolic syndrome are associated with periodontal disease^15,30^. The development of obesity promotes the circulating level of inflammatory proteins secreted from adipocytes and adipose tissue-derived macrophages, which induces systemic inflammation^31^. Our data shows that the effect of PLAP-1 on alveolar bone resorption is distinct between NC and HFD feeding. We speculated that HFD-fed KO mice are less obese and metabolically more healthy, leading to less bone resorption. It is interesting to investigate the PLAP-1 expression in PDL during HFD feeding for future study.

We found that PLAP-1 cell-autonomously enhances adipocyte differentiation from preadipocytes to mature adipocytes in 3T3-L1 cells. PLAP-1 is known to bind TGF-β and suppress TGF-β-induced chondrogenesis^32^. Adipocyte differentiation is promoted by suppressing TGF-β expression in 3T3-L1 cells. It is suggested that TGF-β may inhibit adipocyte differentiation^33^. Thus, whether PLAP-1 promotes adipocyte differentiation through suppression of TGF-β signaling will need to be addressed in the future.

In subcutaneous adipose tissue, PLAP-1 is mainly expressed in SVF containing preadipocytes, endothelial cells, macrophages, and fibroblasts^34^. This data suggests that PLAP-1 may be expressed not only in preadipocytes but also in mesenchymal stem/progenitor cells in adipose tissue and may be involved in maintaining adipose tissue homeostasis through association with other ECM. Collagen I, III, and VI are reportedly involved in fibrosis of adipose tissue^22^, and the absence of collagen VI results in improved metabolism^23^. We demonstrated that *Col6a1* expression was decreased in the SVF of *Plap-1* KO mice. The decreased collagen VI expressions may have improved the metabolism of *Plap-1* KO mice. Furthermore, *Dcn* and *Bgn* expressions were significantly lower in the SVF of *Plap-1* KO mice. PLAP-1 may promote fibrosis by affecting the expression of other SLRPs.

In adipose tissue fibrosis, adipocytes surrounded by ECM undergo necrosis, and M1 macrophages infiltrate the adipose tissue and cause inflammation^35^. Macrophages produce inflammatory cytokines, such as TNF-α, and cause insulin resistance^36^. In adipose tissue of *Plap-1* KO mice, macrophage marker gene expression was lower compared to WT mice, suggesting that decreased recruitment of macrophages and decreased production of inflammatory cytokines may improve insulin resistance in adipose tissue of *Plap-1* KO mice. PLAP-1 is expressed in cancer-associated fibroblasts of scirrhous gastric cancer and promotes invasion through activation of the CD44-Rac1 pathway^37^. PLAP-1 may promote the infiltration of macrophages mediated by CD44 activation. Interestingly, adipose tissue macrophage-derived Biglycan is recently reported to activate inflammatory genes in adipocytes^38^. Further study is needed to determine whether macrophage secretes other SLRPs, including PLAP-1.

Triglyceride in the blood is taken into adipocytes as lipid droplets through the activation of LPL in endothelial cells^39,40^. Interestingly, *Plap-1* KO showed significantly higher serum concentration of triglyceride than WT mice. This result suggests that the activation of LPL is low in adipose tissue of *Plap-1* KO mice and increases the serum concentration of triglycerides. Binding of LPL to LDL receptor-related protein-1 (LRP) promotes the uptake of triglyceride into adipocytes^41^ and Decorin regulates TGF-β signaling by binding the region of leucine-rich repeat to LRP^42^. Similarly, PLAP-1 may influence the activation of LPL through LRP.

Human aspartic acid (D) repeat polymorphisms in its N-terminal region of PLAP-1 are different individually^32^. We demonstrated that D14-PLAP-1 suppressed BMP-2 signaling more efficiently than D13-PLAP-1 through binding with BMP-2^43^. Genetic polymorphism of PLAP-1 in adipose tissue may influence the development of obesity, adipocyte differentiation, and periodontal health.

In conclusion, we demonstrated that the absence of PLAP-1 inhibited HFD-induced metabolic disorder and alveolar bone resorption in vivo, and adipocyte differentiation with the change of other ECM expression in vitro. PLAP-1 may provide insights into the association between diabetes and periodontal disease.

## Supplementary Information

Supplementary Information 1.

## Data Availability

All data needed to evaluate the conclusions in the paper are present in the paper and/or the Supplementary Materials. Additional data related to this paper may be requested from the authors.
