# Supplementary material for: Mice lacking PLAP-1/asporin counteracts high fat diet-induced metabolic disorder and alveolar bone loss by controlling adipose tissue expansion
Source: Sci Rep. 2021 Mar 2;11:4970. doi: 10.1038/s41598-021-84512-2 (PMC7925592; doi:10.1038/s41598-021-84512-2)
Supplement: Supplementary file 1 — Supplementary Information 1. [file 41598_2021_84512_MOESM1_ESM.pdf]

Mice lacking PLAP-1/asporin counteracts high fat diet-induced metabolic disorder and alveolar bone loss by controlling adipose tissue expansion

Hiromi Sakashita[1], Satoru Yamada[1,2*], Masaki Kinoshita[1], Tetsuhiro Kajikawa[1,#a], Tomoaki Iwayama[1], and Shinya Murakami[1*]

[1] Department of Periodontology, Osaka University Graduate School of Dentistry, Suita, Osaka, Japan

[2] Department of Periodontology and Endodontology, Tohoku University Graduate School of Dentistry, Sendai, Miyagi, Japan

[#a] Current Address: Department of Basic and Translational Sciences, Laboratory of Innate Immunity and Inflammation, Penn Dental Medicine, University of Pennsylvania, Philadelphia, Pennsylvania, United States of America

* Corresponding author

E-mail: satoruy@tohoku.ac.jp (SY) and ipshinya@dent.osaka-u.ac.jp (SM)

Fig S1

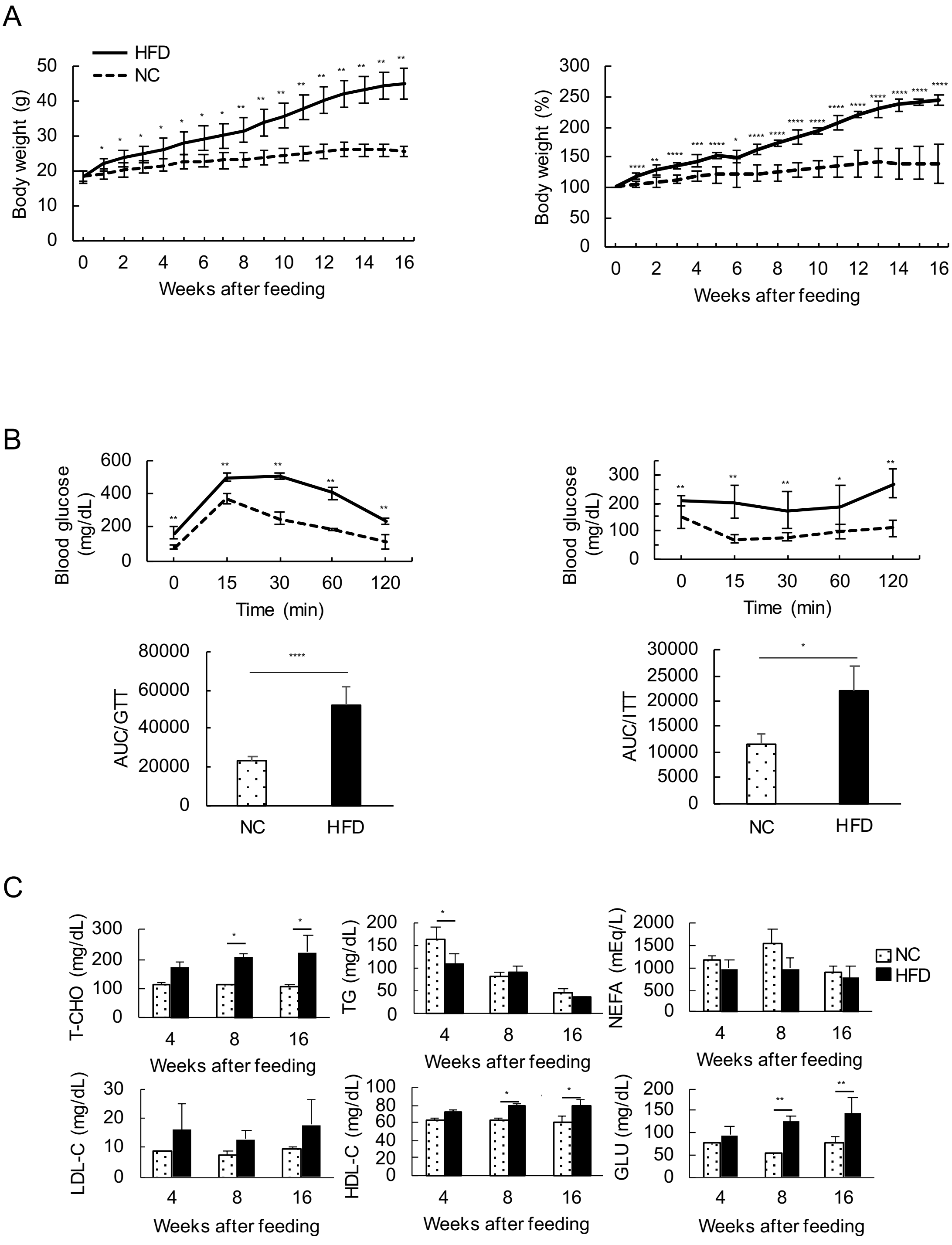

A
HFD
NC
Body weight (g)
Weeks after feeding
Body weight (%)
Weeks after feeding
B
Blood glucose (mg/dL)
Time (min)
Blood glucose (mg/dL)
Time (min)
AUC/GTT
NC
HFD
AUC/ITT
NC
HFD
C
T-CHO (mg/dL)
Weeks after feeding
TG (mg/dL)
Weeks after feeding
NEFA (mEq/L)
Weeks after feeding
NC
HFD
LDL-C (mg/dL)
Weeks after feeding
HDL-C (mg/dL)
Weeks after feeding
GLU (mg/dL)
Weeks after feeding

Fig S1. HFD-induced obesity and metabolic abnormalities.

(A) 5-week-old male C57BL/6J mice were fed with high fat diet (HFD) or normal chow diet (NC) and weighted weekly (n = 7 in each group). (B) Glucose tolerance test (GTT) and insulin tolerance test (ITT) were performed in mice after NC or HFD feeding (n = 4 in each group). (C) Serum levels of total cholesterol (T-CHO), triglyceride (TG), LDL-cholesterol (LDL-C), HDL-cholesterol (HDL-C), non-esterified fatty acid (NEFA), glucose (GLU) were measured during NC or HFD feeding (n = 3 in each group). *: $p < 0.05$, **: $p < 0.01$, ****: $p < 0.0001$.

Fig S2

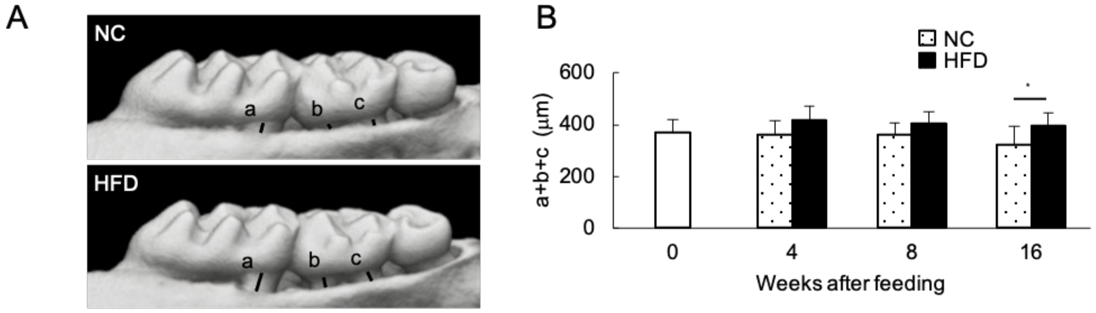
A
NC
a
b
c
HFD
a
b
c
B
NC
HFD
600
400
200
0
a+b+c (μm)
*
0
4
8
16
Weeks after feeding

Fig S2. HFD-induced alveolar bone resorption.

(A) Alveolar bone resorption in mice fed with NC or HFD was evaluated by μCT. (B) Distance between alveolar bone crest and cement-enamel junction was measured at distal root of first molar (a), mesial (b) and distal (c) root of second molar (n = 14 in each group). Results show the mean ± SD. *: $p < 0.05$

Fig S3

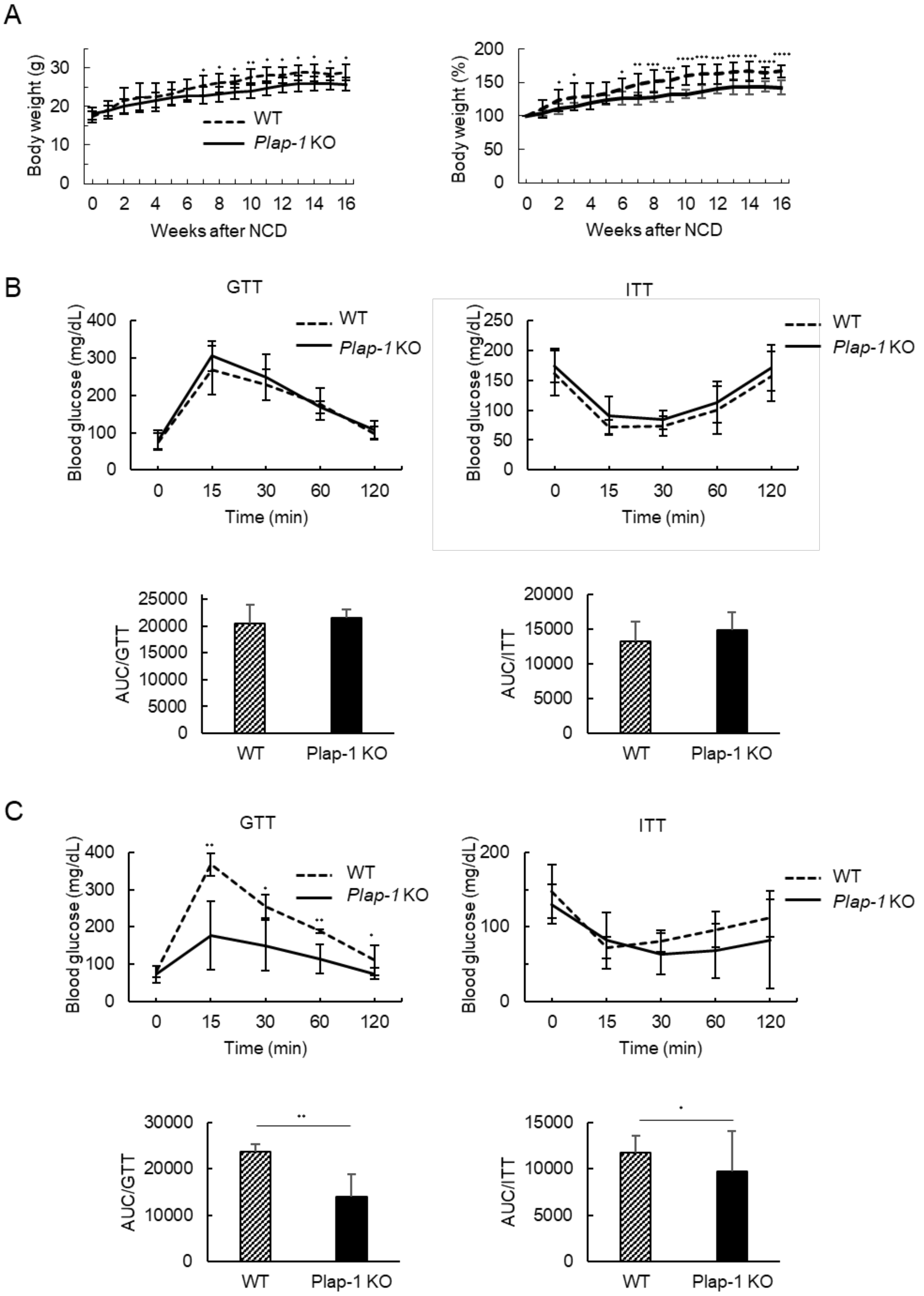

A
Body weight (g)
Weeks after NCD
WT
Plap-1 KO
Body weight (%)
Weeks after NCD
B
GTT
Blood glucose (mg/dL)
Time (min)
WT
Plap-1 KO
ITT
Blood glucose (mg/dL)
Time (min)
WT
Plap-1 KO
AUC/GTT
WT
Plap-1 KO
AUC/ITT
WT
Plap-1 KO
C
GTT
Blood glucose (mg/dL)
Time (min)
WT
Plap-1 KO
ITT
Blood glucose (mg/dL)
Time (min)
WT
Plap-1 KO
AUC/GTT
WT
Plap-1 KO
AUC/ITT
WT
Plap-1 KO

Fig S3. Body weight change and glucose homeostasis in *Plap-1* KO mice with NC feeding.

(**A**) Body weight changes in WT and *Plap-1* KO mice during NC feeding. 5-week-old male WT and *Plap-1* KO mice were fed with NC and weighted weekly. WT (n = 11), *Plap-1* KO (n = 8). Results show the mean ± SD. (**B**) GTT and ITT were performed in 5-week-old male WT and *Plap-1* KO mice (n = 9 in each group). (**C**) GTT and ITT were performed in WT and *Plap-1* KO mice after 16 weeks NC feeding. For GTT, WT (n = 8), *Plap-1* KO (n = 7) and for ITT, WT (n = 8), *Plap-1* KO (n =8). Results show the mean ± SD. *: $p < 0.05$, **: $p < 0.01$, ***: $p < 0.001$, ****: $p < 0.0001$.

Fig S4

A

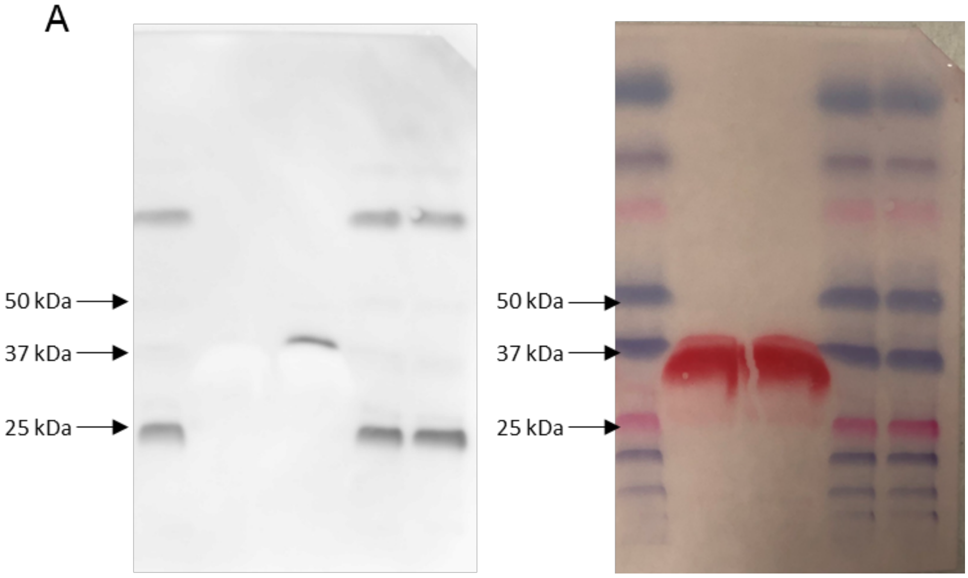

Fig S3. Immunoblots performed in this study

(**A**) anti-FLAG blot (left) and Ponceau S staining (right).

Table S1

Gene of interest		Forward primers sequences	Reverse primers sequences
Aspn	NM_025711	atgatgacgataacgatgatgacga	tgttgtttggaaccgatgtcaga
Adipoq	NM_009605	ttctgtctgtacgattgtcagtgga	ggcatgactgggcaggatta
Lep	NM_008493	gaacctgtctactcatgccagcac	ctggtcctgcagcctgtttg
Adgre1	NM_010130	gctatgggcttccagtcctggtggt	gaaggtcagcaacctcgtgtccttg
Tnf	NM_013693	aagcctgtagcccacgtcgta	ggcaccactagttggttgtctttg
Il6	NM_031168	ccacttcacaagtcggaggctta	gcaagtgcatcatcgttgttcatac
Ccl3	NM_011337	catgacactctgcaaccaagtcttc	gagcaaaggctgttggtttca
Arg1	NM_007482	agctctgggaatctgcatgg	atgtacacgatgtctttggcagata
Chil3	NM_009892	gatggcctcaacctggactg	cgtcaatgattcctgctcctg
Clec7a	NM_001309637	ggtaccctgactcaaatttcacaac	caccacagtaagtaggctgattctc
Fabp4	NM_024406	tgggaacctggaagcttgtctc	gaattccacgcccagtttga
Pparg	NM_011146	ggagcctaagtttgagtttgctgtg	tgcagcaggttgtcttggatg
Cebpa	NM_007678	ttgaagcacaatcgatccatcc	gcacactgccattgcacaag
Col1a1	NM_007742	cagggtattgctggacaacgtg	ggaccttgtttgccaggttca
Col3a1	NM_009930	caggccagtggcaatgtaaaga	ctcattgccttgcgtgtttgata-
Col6a1	NM_009933	gagcttctcgatgacggctttc	gagctgtctagcaggatggtgatg
Dcn	NM_007833	ctgggctggcacagcataagta	cggacagggttgccgtaaag
Bgn	NM_007542	gatgattgagaatgggagcctga	tccgaagcccataggacagaag
Hprt	NM_013556	ttgttgttggatatgcccttgacta	aggcagatggccacaggacta
